# Response certainty during bimanual movements reduces gamma oscillations in primary motor cortex

**DOI:** 10.1016/j.neuroimage.2020.117448

**Published:** 2020-10-12

**Authors:** Alex I. Wiesman, Nicholas J. Christopher-Hayes, Jacob A. Eastman, Elizabeth Heinrichs-Graham, Tony W. Wilson

**Affiliations:** a Department of Neurological Sciences, University of Nebraska Medical Center, 988422 Nebraska Medical Center, Omaha, NE 68198-8422, USA; b Center for Magnetoencephalography, UNMC, Omaha, NE, USA; c Cognitive Neuroscience of Development & Aging (CoNDA) Center, UNMC, Omaha, NE, USA

## Abstract

Even when movement outputs are identical, the neural responses supporting them might differ substantially in order to adapt to changing environmental contexts. Despite the essential nature of this adaptive capacity of the human motor system, little is known regarding the effects of contextual response (un)certainty on the neural dynamics known to serve motor processing. In this study, we use a novel bimanual motor task and neuroimaging with magnetoencephalography (MEG) to examine the effects of contextual response certainty on the dynamic neural responses that are important for proper movement. Significant neural responses were identified in the time-frequency domain at the sensor-level and imaged to the cortex using a spectrally resolved beamformer. Combined frequentist and Bayesian statistical testing between neural motor responses under certain and uncertain conditions indicated evidence for no conditional effect on the peri-movement beta desynchronization (18 – 28 Hz; −100 to 300 ms). In contrast, the movement-related gamma synchronization (MRGS; 66 – 86 Hz; −50 to 150 ms) exhibited a robust effect of motor certainty, such that increased contextual response certainty reduced the amplitude of this response. Interestingly, the peak frequency of the MRGS was unaffected by response certainty. These findings both advance our understanding of the neural processes required to adapt our movements under altered environmental contexts, and support the growing conceptualization of the MRGS as being reflective of ongoing higher cognitive processes during movement execution.

## 1. Introduction

As our senses sample stimuli from our environment and allow us to build predictive internal models of the world around us, our motor system enables us to act and react within the context of these predictions. The online process of integrating sensory input and motor output is extremely dynamic and thought to be implemented through a complex series of systems-level neural interactions that unfold on a sub-second timescale. One of the most important features of human cognitive-sensorimotor interactions is the ability to adapt to differing levels of contextual certainty. In other words, in situations where the need to produce a motor output is uncertain, the neural systems serving these outputs must flexibly adapt to maintain accurate motor performance. Despite the pervasive nature of such uncertainty, very little research has focused on delineating the involved neural systems and their dynamics.

Extant research has established that motor function requires the recruitment of neural populations along the precentral gyrus, and that the representation of different body parts along this gyrus follows a stereotyped homuncular organization (Cheyne et al., 1991; Yousry et al., 1997). Beyond the anatomical definitions of motor circuitry, the temporal and spectral features have also received substantial attention. Studies of the essential role of rhythmic neural activity in the planning and execution of movements have found that multi-spectral and temporally-defined responses support distinct aspects of motor function, even when they occur in spatially-overlapping regions (Cheyne et al., 2008; Heinrichs-Graham et al., 2016; Heinrichs-Graham et al., 2018; Heinrichs-Graham et al., 2017; Heinrichs-Graham and Wilson, 2015, 2016; Jurkiewicz et al., 2006; Pfurtscheller, 2000; Pfurtscheller and Lopes da Silva, 1999; Wiesman et al., 2020; Wilson et al., 2014; Wilson et al., 2010). Perhaps most auspiciously, a strong event-related desynchronization (ERD; i.e., a decrease from baseline levels of synchronous neural activity) is usually observed in the beta (15 – 30 Hz) band, beginning shortly before motor onset and extending through movement. Numerous studies have shown that this response is essential for both the planning and execution of movements (Doyle et al., 2005; Grent-’t-Jong et al., 2014; Heinrichs-Graham et al., 2016; Heinrichs-Graham and Wilson, 2015; Kaiser et al., 2001; Pogosyan et al., 2009; Praamstra et al., 2009; Tzagarakis et al., 2010), and is thought to represent a general dis-inhibition of resting motor network synchrony in order to facilitate motor performance. A short-lived event-related synchronization (ERS) in the gamma (30+ Hz) band is also often found to be temporally overlapping with the beta response (Cheyne et al., 2008; Wilson et al., 2010), however the functional importance of the so-called movement-related gamma synchronization (MRGS) is less well-known. Early research indicated that the gamma ERS might be a non-specific marker of movement execution, thus implying that this response would be insensitive to changes in cognitive state and/or cognitive dimensions of the stimuli (Cheyne and Ferrari, 2013). However, in recent years this interpretation has been challenged by work showing that both the amplitude and peak frequency of the gamma ERS is modulated by cognitive interference (Gaetz et al., 2013; Grent-’t-Jong et al., 2013; Heinrichs-Graham et al., 2018; Isabella et al., 2015; Wiesman et al., 2020). Despite this growing literature, many questions regarding the nature of the MRGS remain, including whether contextual response certainty might exert an effect on this response. Finally, a resynchronization in the beta band commonly occurs well after movement offset, beginning roughly 500 ms after movement and continuing for up to 2000 ms thereafter (Gaetz et al., 2010; Heinrichs-Graham et al., 2017; Jurkiewicz et al., 2006; Parkes et al., 2006; Trevarrow et al., 2019; Wilson et al., 2010). Importantly, this response has been implicated in the termination of motor output (Heinrichs-Graham et al., 2017), and is thus more likely to be important for sensory feedback and/or active inhibition than movement execution.

In this study, we examine the effect of contextual response certainty on the oscillatory dynamics serving motor control by holding the motor response constant across conditions while varying the probability that a response would be required. To this end, we recorded and analyzed magnetoencephalography (MEG) data from 25 healthy right-handed young adults as they performed a novel bi-manual motor task, wherein they were infrequently required to inhibit responses from one of their two hands. This task design provided a within-participant conditional contrast that varied in terms of contextual response certainty. In light of recent literature highlighting the impact of cognitive state on the MRGS, we hypothesized that both the amplitude and peak frequency of this response would be modulated by response certainty. In contrast, since the beta ERD is thought to be more strongly associated with motor planning, we did not expect that it would be significantly affected.

## 2. Materials and methods

### 2.1. Participants

We enrolled 25 healthy young adults (mean age = 24.90 years; SD = 3.49 years; range = 19.84 - 33.80 years; 15 males/10 females; all right handed) for participation in this study. Exclusionary criteria included any medical illness affecting CNS function, any neurological disorder, history of head trauma, any non-removable metal implant that would adversely affect data acquisition, and current substance abuse. The Institutional Review Board at the University of Nebraska Medical Center reviewed and approved this investigation. After complete description of the study, written informed consent was acquired from each participant. All participants had normal or corrected-to-normal vision and completed the same experimental protocol.

### 2.2. Experimental paradigm

**Fig. 1.**
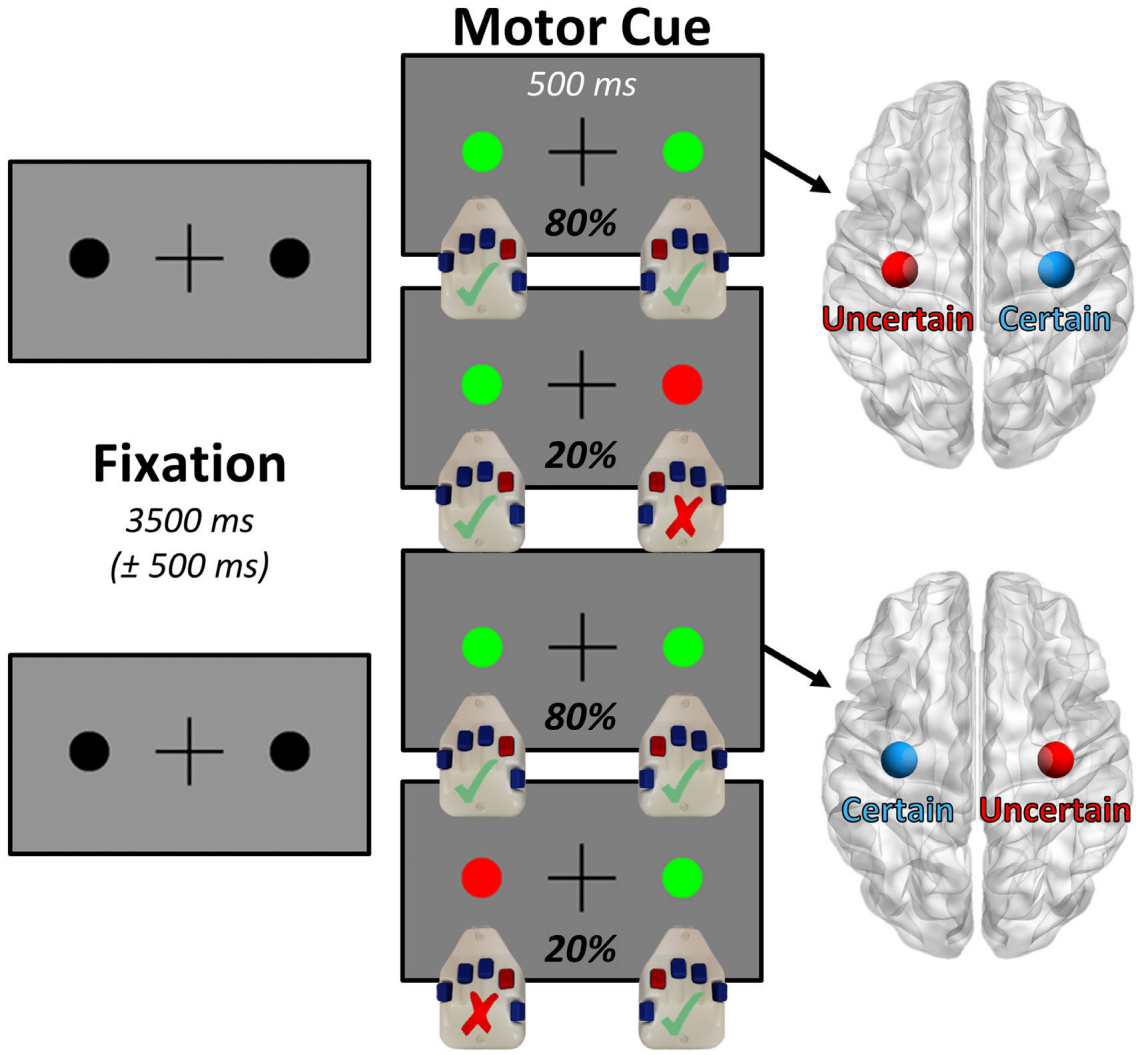
Bimanual motor task design. Participants completed 150 trials of the paradigm shown above, each of which began with a variable fixation period (3500 ± 500 ms), a portion of which functioned as our pre-movement baseline. Following this fixation period, each of the two black circles flanking the central fixation cross would change color (500 ms), with two possible stimulus combinations per participant. If both of the circles turned green (80% of trials), the participant was instructed to respond by button press with their index finger on both hands, and importantly these trials were the only ones included in further analysis. In contrast, if one of the two circles turned red (consistently on the left or right side, counterbalanced across participants), then the participant was told to inhibit the motor response on the hand corresponding to that side. This created a lateralized effect of contextual motor certainty, allowing us to contrast the amplitude of the bilateral motor responses representing the bimanual movements within participants to examine effects of motor certainty on motor-related oscillatory dynamics.

Participants were seated in a custom-made nonmagnetic chair with their head positioned within the MEG sensor array. During the scan, participants performed a bimanual motor paradigm, wherein they were infrequently required to inhibit responses from one of their two hands, which provided a within-participant conditional contrast that varied in terms of contextual response certainty (Fig. 1). The task began with a visual fixation period consisting of a centrally presented crosshair flanked on both sides by small black circles, the duration (3500 ms) of which was randomly jittered (± 500 ms) to avoid any neural effects of expectation. After the end of the fixation period, each of the two black circles changed color to either green or red for a fixed period of 500 ms. Prior to the start of the task, the participants were instructed that this color change would indicate whether they should press a button with their index finger on the hand corresponding to that side of the screen. A green circle indicated that they should press the corresponding button, while a red circle indicated that the motor response should instead be inhibited. Across the entire 150 trials completed by each participant, only one of the two black circles (i.e., on the left or right) had the potential to turn red, meaning that each participant was essentially performing a simple cued motor task with one hand (high response certainty), and a cued Go/No-Go motor task with the other (low response certainty). The hand chosen for the inhibitory red motor cues was counterbalanced across the participants, so as to null any potential effects of hand dominance. Importantly, any trial containing a red “inhibit” cue (20% of trials; pseudo-randomized) was excluded from further analysis, and only trials where the participant responded correctly with both hands were included. By analyzing only the dual-response trials (80% of trials), we were able to statistically contrast bilateral primary motor responses within each participant, and thus compare the effect of contextual response certainty on these neural dynamics.

Total MEG recording time was approximately 10 minutes per participant. Custom visual stimuli were programmed in Matlab (Math-works, Inc., Massachusetts, USA) using Psychophysics Toolbox Version 3 (Brainard, 1997) and back-projected onto a semi-translucent non-ferromagnetic screen at an approximate distance of 1.07 meters, using a Panasonic PT-D7700U-K model DLP projector with a refresh rate of 60 Hz and a contrast ratio of 4000:1.

### 2.3. MEG data acquisition

Our MEG data acquisition, structural coregistration, preprocessing, and sensor-/source-level analyses closely followed the analysis pipeline of previous manuscripts (Heinrichs-Graham et al., 2018; Wiesman et al., 2018; Wiesman et al., 2017; Wiesman et al., 2018; Wiesman and Wilson, 2019; Wiesman and Wilson, 2019b; Wiesman and Wilson, 2020). All recordings were conducted in a one-layer magnetically-shielded room with active shielding engaged for environmental noise compensation. Neuromagnetic responses were sampled continuously at 1 kHz with an acquisition bandwidth of 0.1– 330 Hz using a 306-sensor Elekta/MEGIN MEG system (Helsinki, Finland) equipped with 204 planar gradiometers and 102 magnetometers. Participants were monitored during data acquisition via real-time audio-video feeds from inside the shielded room. Each MEG dataset was individually corrected for head motion and subjected to noise reduction using the signal space separation method with a temporal extension (correlation limit: .950; correlation window duration: 6 seconds; Taulu and Simola, 2006). Only data from the gradiometers were used for further analysis.

### 2.4. Structural MRI processing and MEG coregistration

Preceding MEG measurement, four coils were attached to the participant’s head and localized, together with the three fiducial points and scalp surface, using a 3-D digitizer (Fastrak 3SF0002, Polhemus Navigator Sciences, Colchester, VT, USA). Once the participant was positioned for MEG recording, an electric current with a unique frequency label (i.e., 293, 307, 314, and 321 Hz) was fed to each of the coils. This induced a measurable magnetic field and allowed each coil to be localized in reference to the sensors throughout the recording session. Since coil locations were also known in head coordinates, all MEG measurements could be transformed into a common coordinate system. With this coordinate system, each participant’s MEG data were co-registered with structural T1-weighted MRI data using BESA MRI (Version 2.0) prior to source-space analysis. Structural MRI data were aligned parallel to the anterior and posterior commissures and transformed into Talairach space. Following source analysis (i.e., beamforming), each participant’s 4.0 × 4.0 × 4.0 mm functional images were also transformed into Talairach space using the transform that was previously applied to the structural MRI volume and spatially resampled.

### 2.5. MEG preprocessing, time-frequency transformation, and sensor-level statistics

Cardiac and blink artifacts were removed from the data using independent component analysis (ICA; Vigário et al., 1998). Principle components (minimum variance: 1%) were decomposed into independent components using an extended infomax algorithm (Lee et al., 1999). Prior to exclusion from the reconstructed data, both the spatial and temporal topography of each component was visually inspected, and only those components exhibiting clear stereotyped spatio-temporal patterns consistent with ocular/cardiac artifacts were excluded. The ICA-corrected continuous magnetic time series was then filtered between 0.5 – 200 Hz (plus a 60 Hz notch filter), and divided into 3500 ms epochs, with zero defined as the button press, and the baseline extending from −1400 to −1000 ms prior to the button press, so as to avoid any contamination of the baseline by preparatory movement responses.

Epochs containing artifacts were rejected using an individualized fixed threshold method, supplemented with visual inspection. Briefly, in MEG, the raw signal amplitude is strongly affected by the distance between the brain and the MEG sensor array, as the magnetic field strength falls off sharply as the distance from the current source increases. To account for this source of variance across participants, as well as actual variance in neural response amplitude, we used an individually-determined threshold based on the signal distribution for both signal amplitude and gradient to reject artifacts. Across all participants, the average amplitude threshold was 1077.24 (SD = 441.91) fT/cm and the average gradient threshold was 192.56 (SD = 153.36) fT/(cm*ms). Across the group, an average of 103.52 (SD = 9.78) dual-response trials per participant (out of 120 possible trials) were used for further analysis. Importantly, none of our statistical comparisons were compromised by differences in trial number nor artifact thresholds, as all of our contrasts of interest were computed between bilateral motor peaks within the same participants and trials.

To investigate the oscillatory responses commonly associated with motor processing, we next transformed the same post-artifact-rejection epochs into the time-frequency domain using complex demodulation (Hoechstetter et al., 2004; Kovach and Gander, 2016; Papp and Ktonas, 1977). Briefly, complex demodulation works by first transforming the signal into the frequency space, using a Fast Fourier Transform (FFT). This results in a frequency spectrum, inherently containing the same power and cross spectrum information as the original signal. From here, this frequency spectrum is (de)modulated in a step-wise manner to adopt the center frequency of a series of complex sinusoids with increasing carrier frequencies, in a process termed "heterodyning." These resulting signals are then low-pass filtered to reduce spectral leakage, and thus the nature of this filter inherently determines the time and frequency resolution of the resulting data. For this study, the time-frequency analysis was performed with a frequency-step of 2 Hz and a time-step of 25 ms between 4 and 100 Hz, using a 4 Hz lowpass finite impulse response (FIR) filter with a full-width half maximum (FWHM) in the time domain of ~115 ms. The resulting spectral power estimations per sensor were averaged over trials to generate time-frequency plots of mean spectral density, which were normalized by the baseline power of each respective bin, calculated as the mean power during the −1400 to −1000 ms time period. The time-frequency windows used for the time-frequency domain source analysis (described below) were determined by means of a paired-sample cluster-based permutation test against baseline across all participants and the entire frequency range (4 – 100 Hz), with an initial cluster threshold of *p* < .05 and 10,000 permutations. To ensure that all responses used in further analysis were oscillatory in nature, we also computed the same sensor-level time-frequency transform with the evoked signal removed using regression, and did not pursue subsequent analysis of any time-frequency cluster that did not remain after this procedure.

### 2.6. MEG source analysis

Time-frequency resolved beamformer source images were computed using the dynamic imaging of coherent sources (DICS; regularization: singular value decomposition .0001%; Gross et al., 2001) approach, which uses the time-frequency averaged cross-spectral density to calculate voxel-wise estimates of neural power. Following convention, we computed noise-normalized, source power per voxel in each participant using active (i.e., task) and passive (i.e., baseline) periods of equal duration and bandwidth. Such images are typically referred to as pseudo-t maps, with units (pseudo-t) that reflect noise-normalized power differences (i.e., active vs. passive) per voxel. For visualization purposes, the resulting images were grand-averaged within each time-frequency response. Due to the spatial variability of individual brain structure and function, as well as the relatively focal nature of primary motor responses, individual peak voxels in the bilateral somatomotor cortices were identified for each time-frequency response per participant. Pre-requisites for peak selection were that the responses had to be (1) within 3.5 cm (Euclidian distance) of the grand-averaged response peak across all participants, (2) distinct peaks with a separable maximum (MRGS) or minimum (beta ERD), and (3) have a contralateral counterpart within the same participant and frequency band. Importantly, the Euclidian distance of these locations from the peak voxel of the grand average response did not significantly differ across our contrast of interest (i.e., uncertain vs. certain; MRGS: *p* = .600; beta ERD: *p* = .700). Response amplitude values (i.e., pseudo-t) were extracted from these peak voxels, and these values were compared statistically for effects of certainty condition. In addition, using these peak voxel locations, virtual sensor data were computed by applying the sensor-weighting matrix derived through the forward computation to the preprocessed signal vector, which yielded a time series corresponding to the location of interest. These virtual sensor data were then decomposed into time-frequency space, which resulted in time-frequency representations for each of the bilateral motor responses in each participant. From these time-frequency data, amplitude envelopes were computed to display the temporal evolution of the differences found in the peak voxel analysis, and peak frequency data were extracted to compare effects of condition on the speed of the underlying neural oscillations.

### 2.7. Statistical analyses and software

To examine the effects of contextual motor certainty on each oscillatory neural response, we computed paired-samples t-tests between the certain and uncertain motor responses (as determined by the laterality of the uncommon “inhibit” cue) within our sample. To complement our initial frequentist statistical approach, Bayesian analysis was also performed in *JASP*, using a zero-centered Cauchy distribution with a default scale of 0.707. All primary data preprocessing, coregistration, and sensor- and source-level analyses were performed in the Brain Electrical Source Analysis software suite (BESA Research v6.1 and BESA MRI v2.0). Cluster-based permutation testing on sensor-array data was performed in BESA Statistics (v2.0), and all parametric and Bayesian statistics were computed in *JASP* (JASP-Team, 2018).

## 3. Results

### 3.1. Task performance and response-locked neural oscillatory responses

Participants generally performed well on the dual-response task (Fig. 1), responding correctly to 77.10% (SD: 11.16%) of the unimanual “oddball” trials and 99.66% (SD: 0.43%) of the bimanual trials. The average reaction time on the task was 373.24 ms (SD: 64.17 ms) for the bimanual trials and 449.16 ms (SD: 101.91 ms) for the unimanual trials. Importantly, the bimanual trials were the focus of all subsequent analysis.

**Fig. 2.**
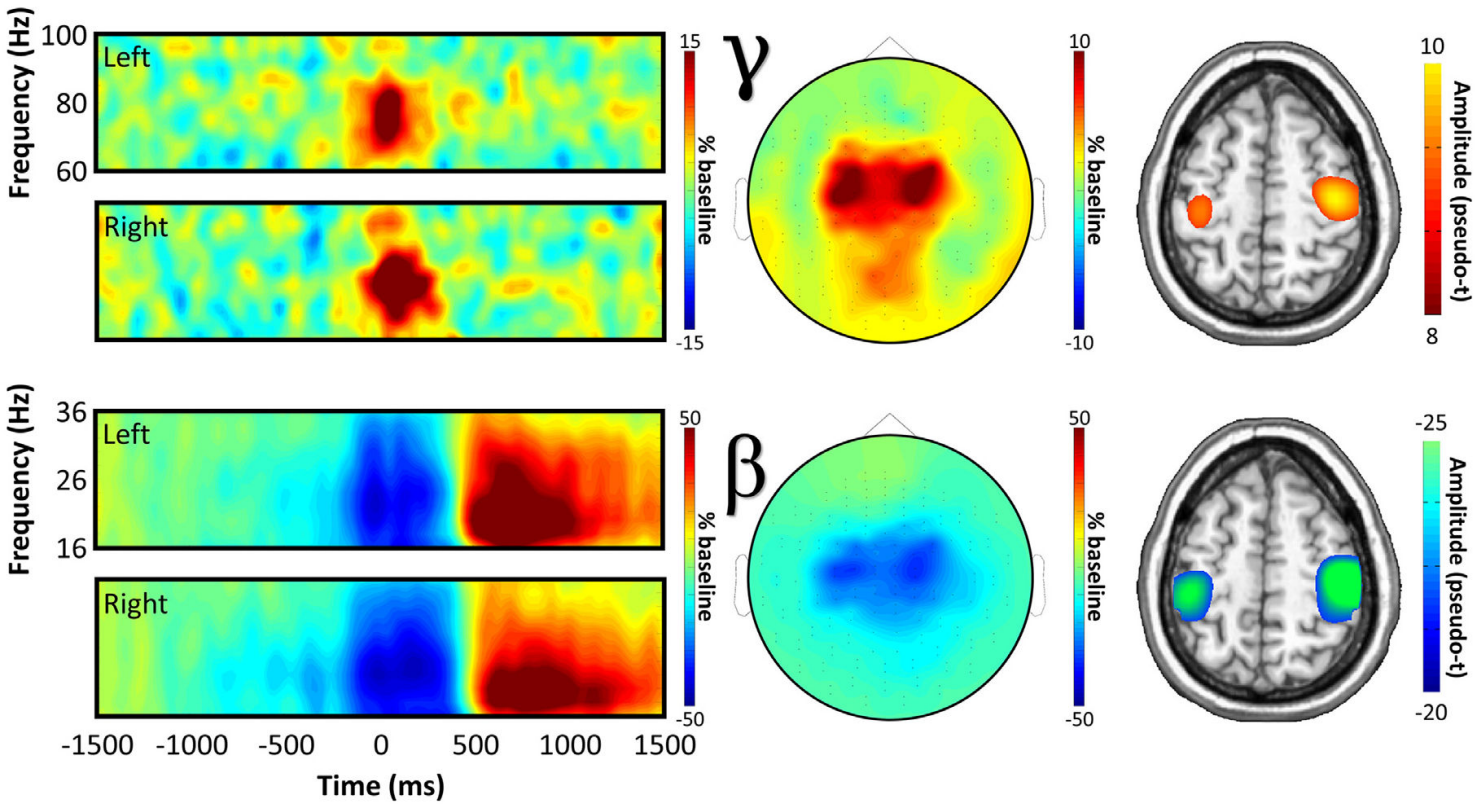
Movement-related neural oscillatory responses. Spectrograms (far left) show grand-averaged data from representative sensors for each bilateral movement-locked neural response, with time (in ms) on the x-axis and frequency (in Hz) on the y-axis. Cluster-based permutation testing indicated significant oscillatory responses in the beta (18 – 28 Hz; −100 to 300 ms peri-movement) and gamma (66 – 86 Hz; −50 to 150 ms peri-movement) bands, the spatial distributions of which are displayed in the topographic plots in the center. On the right, spectrally-resolved source images of each response are shown, averaged across all participants.

Prior to testing for conditional effects of response certainty, we first needed to determine the temporal, spectral, and spatial locations of motor-related neural responses to the task, regardless of condition. To this end, we transformed the data into time-frequency space, and observed robust neural activity in the beta and gamma bands (Fig. 2) in sensors near the sensorimotor cortices. Specifically, we observed a significant desynchronization in the beta band (18 – 28 Hz), which ranged temporally from about 100 ms prior to movement to 300 ms after (*beta ERD*; *p* < .001, corrected), as well as a significant synchronization in the gamma band (66 – 86 Hz) that ranged from about 50 ms before movement to 150 ms after (*MRGS*; *p* < .001, corrected). For full time-frequency cluster extents see Fig. S1. Spectrally resolved source imaging revealed that both of these responses originated from the bilateral hand-knob region of the precentral gyrus, commonly referred to as the primary motor cortex. Note that we did not image the post-movement beta rebound response (later ERS in bottom spectrogram of Fig. 2), as we were primarily interested in uncertainty effects on the planning and execution of movement, and the timing of this response (700-1200 ms) was after movement offset making it unlikely to play a key role. Finally, a robust increase in amplitude was also observed in the theta band (4 – 8 Hz; −100 to 150 ms), but this response was no longer significant after removal of the evoked signal. Since this low-frequency cluster was likely a contamination of the time-frequency decomposition by the time-domain evoked response, it was not included in subsequent analyses.

### 3.2. Effects of response certainty on motor-related neural oscillatory responses

**Fig. 3.**
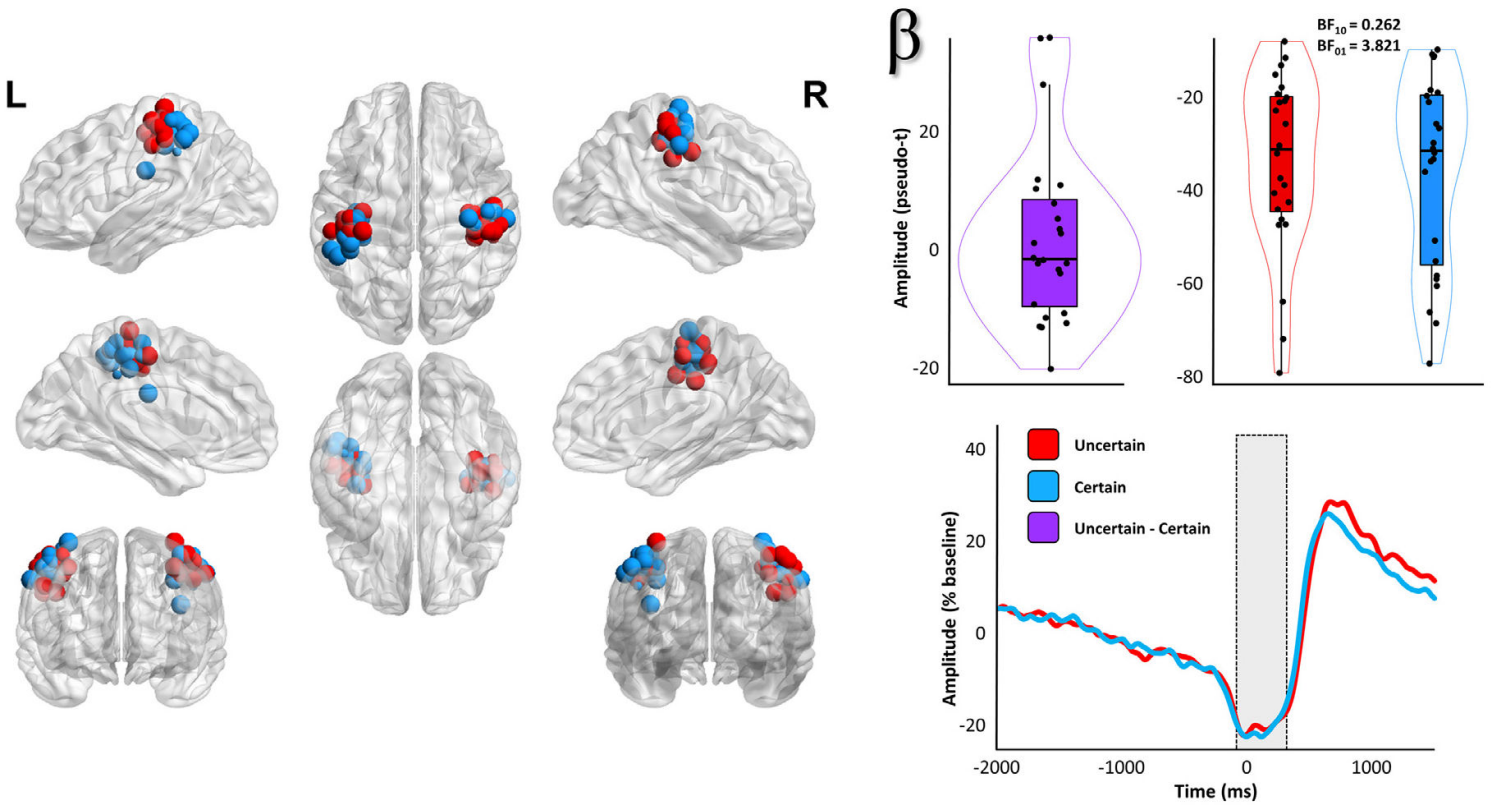
Evidence for no effect of response certainty on the beta ERD. Each point on the images to the left indicates the spatial location (placement of sphere) and relative amplitude (size of sphere) of an individually identified primary motor response peak, with color indicating whether the peak corresponded to the hand where movement was certain (blue) or uncertain (red). To the right of these maps, the participant-level amplitude data subtracted across conditions (top middle) and per condition (top right) are shown. Box plots represent conditional means, first and third quartiles, and minima and maxima, and violin plots show the probability density. The time courses on the bottom right are grand-averaged amplitude envelopes (18 – 28 Hz) extracted from peak voxel virtual sensor data using each participant-specific beta ERD peak, and show the temporal evolution of the (lack of) uncertainty effect. The shaded box indicates the time window identified in the initial sensor-level analysis.

**Fig. 4.**
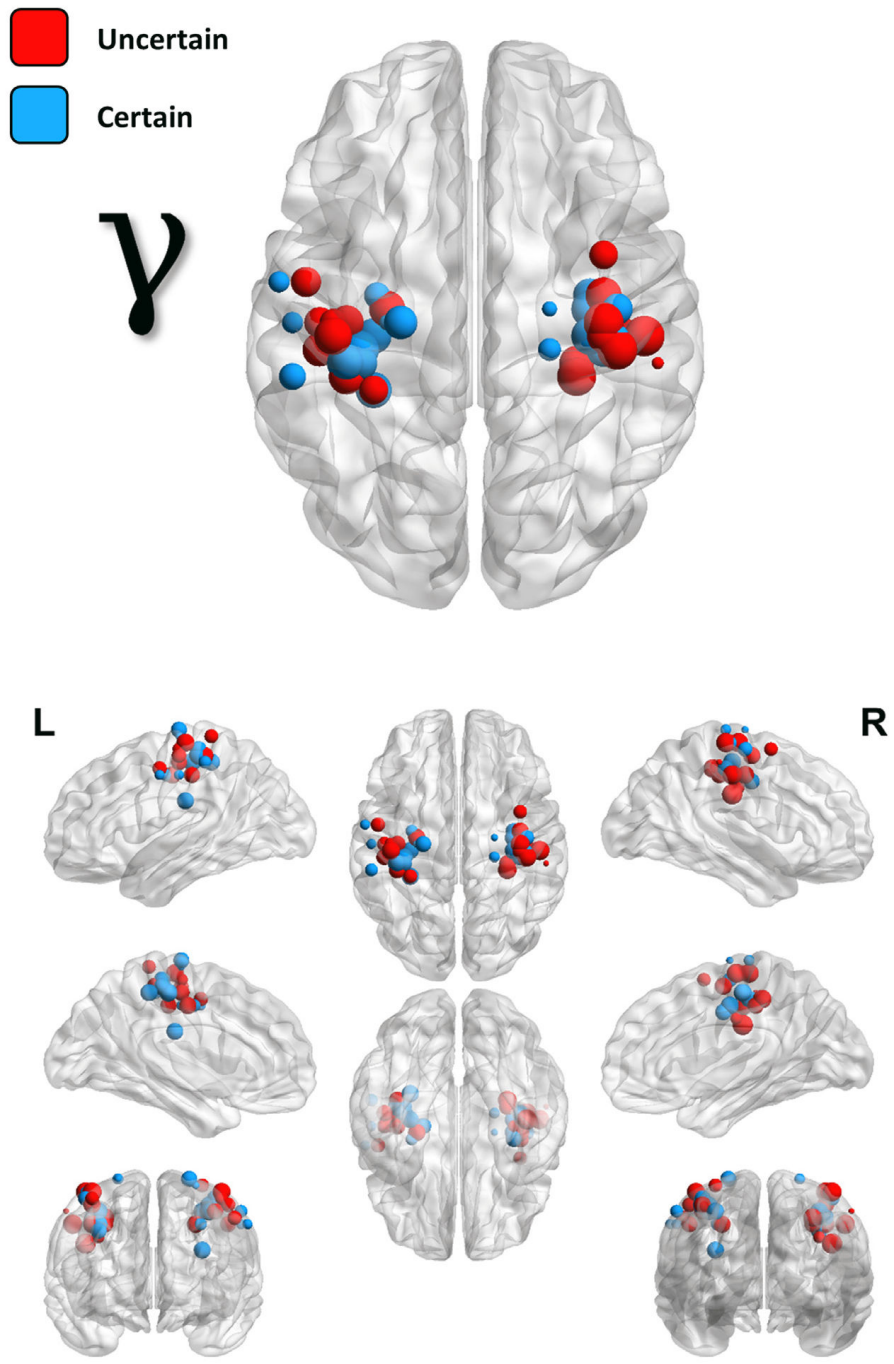
Locations and amplitudes of individual MRGS peaks. Similarly to Fig. 3, each point on these images indicates the spatial location (placement of sphere) and relative amplitude (size of sphere) of individually identified primary motor response peaks, with color indicating whether the peak corresponded to the hand where movement was certain (blue) or uncertain (red).

Once we had identified our neural responses of interest and verified that they were of a motor origin, we then examined the effects of response certainty, manipulated in this study as a function of response laterality (counterbalanced across participants), on these motor oscillations. Importantly, due to the high spatial variability of individual patterns of brain structure and function, as well as the relatively focal nature of primary motor responses (and in particular the MRGS), individual peak voxels in the bilateral somatomotor cortices were identified for each time-frequency response per participant (Figs. 3-4).

**Fig. 5.**
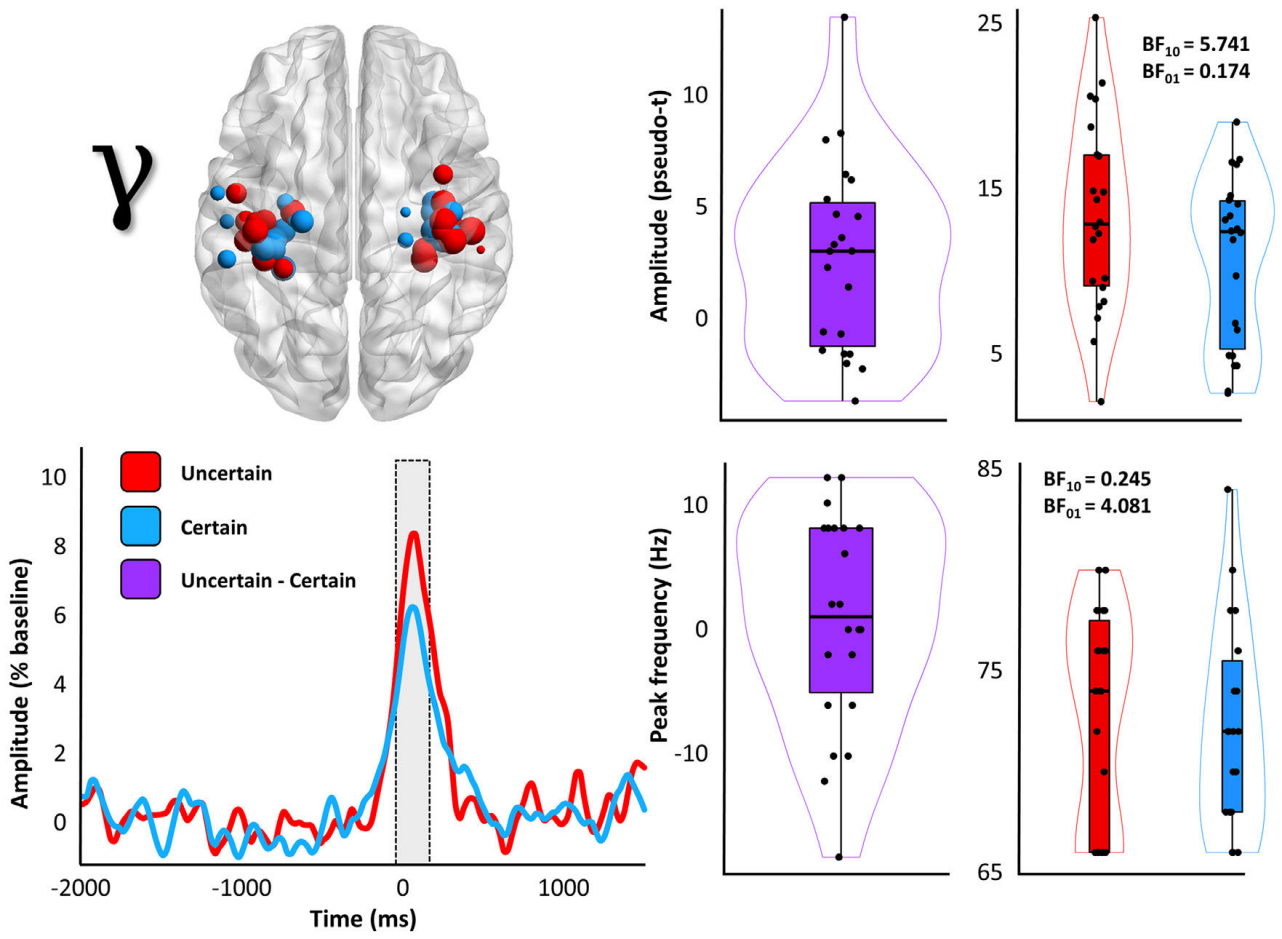
Response certainty reduces the amplitude, but not frequency, of the MRGS. The image on the top left is repeated from Fig. 4, to enhance interpretation. To the right of this map, participant-level amplitude data subtracted across conditions (middle) and per each condition (right) is shown. Box plots represent conditional means, first and third quartiles, and minima and maxima, and violin plots show the probability density. The time courses on the bottom left are grand-averaged amplitude envelopes (66 – 86 Hz) extracted from peak voxel virtual sensor data using each participant-specific MRGS peak, and show the temporal evolution of the uncertainty effect. The shaded box indicates the time window identified in the initial sensor-level analysis. To the right of this plot are the participant-level peak frequency data extracted from these same virtual sensor data, both subtracted across conditions (middle), as well as per each condition (right).

Two-tailed paired-samples t-tests revealed no significant effect of response certainty on the beta ERD (*t*(23) = 0.66, *p* = .515, 95% CI = [−0.27 0.54]; n = 24; Fig. 3) response, and post-hoc Bayesian testing indicated relatively strong evidence for the null hypothesis of no conditional difference (BF_01_ = 3.82). In stark contrast, the MRGS exhibited a robust effect of response certainty (*t*(21) = 2.91, *p* = .008, 95% CI = [0.16 1.07]; n = 22; BF_10_ = 5.74; Fig. 5). To better visualize the temporal dynamics of this effect, we also extracted the amplitude envelope from peak-voxel virtual sensor time series computed using the participant-specific beta ERD and MRGS peaks (Fig. 3, bottom right; Fig. 5, bottom left). It should be noted, however, that all response amplitude statistical comparisons were made using the beamformer output values (i.e., the pseudo-t values). Importantly, neither of these findings were altered when outliers were excluded listwise across both frequencies (*p*_beta ERD_ = .988; *p*_MRGS_ = .008; n = 22). Finally, since previous research has linked the amplitude of the MRGS to the peak frequency of this response, we compared the frequency at which the MRGS exhibited the strongest response amplitude between the two conditions, and found moderate evidence for the null hypothesis of no conditional difference (*t*(21) = 0.46, *p* = .653, 95% CI = [−.32 .52]; n = 22; BF_01_ = 4.08; Fig. 5, bottom right).

## 4. Discussion

Adaptive and dynamic neural systems are critical to support effective motor control in the face of changing environmental contexts. One such context that is ubiquitous in daily life is the need to rapidly and flexibly facilitate or inhibit motor output based on unpredictable environmental cues. In this study, we examined the effect of such contextual response uncertainty on the neural oscillatory dynamics that are known to support motor function in the human brain. Our results indicated that the MRGS was specifically impacted by response certainty, such that enhanced contextual response certainty elicited MRGS responses that were reduced in amplitude. Importantly, this effect was unique to MRGS amplitude, as response certainty did not impact MRGS frequency in this study. This is particularly interesting, as MRGS peak frequency is known to be impacted by other forms of cognitive motor influences (Heinrichs-Graham et al., 2018). This could potentially indicate separable mechanisms of the frequency and amplitude of the induced MRGS response; however, future studies and comprehensive reviews are required to better understand this distinction. Of note, research focusing on visual oscillations in occipital cortex has shown an inverse correlation between peak gamma frequency and amplitude (Campbell et al., 2014; Kujala et al., 2015; Lozano-Soldevilla et al., 2014; Wilson et al., 2018), which suggests the two parameters are linked in other neural systems. In contrast, the beta response did not exhibit a significant effect of response certainty, and in fact, post-hoc Bayesian testing provided support for the null hypothesis for this response. In concert, these findings support a growing recognition that the role of the MRGS is more nuanced than a simple binary signal for motor execution, and is impacted by a number of “higher-order” cognitive factors. Indeed, the MRGS may reflect the certainty or confidence of even strongly stereotyped motor actions (e.g., an index-finger button press).

The MRGS was originally thought to represent a simple reflection of movement execution (Cheyne et al., 2008; Cheyne and Ferrari, 2013; Wilson et al., 2010), and was not expected to be modulated by higher-order cognitive processes such as cognitive interference and response certainty. However, recent lines of research have supported the important role of the MRGS in cognitive-motor integration. Specifically, previous work has shown that multiple types of cognitive interference, including stimulus-stimulus (Gaetz et al., 2013; Grent-’t-Jong et al., 2013; Heinrichs-Graham et al., 2018; Wiesman et al., 2020) and stimulus-response (Wiesman et al., 2020) conflict, can impact the amplitude and/or frequency of the MRGS. Despite these advances, our understanding of the functional significance of the MRGS for proper movement remains underdeveloped. Our finding of an effect of response certainty on the MRGS builds on the conception that it is important for adaptive motor function in concert with ongoing cognition, particularly when the need for motor output is not pre-determined. Additionally, since we found that even just the contextual *possibility* of responding can influence the amplitude of the MRGS, our work provides new evidence that such contextual differences should be carefully balanced in future studies of this neural response.

The finding that response certainty did not impact the beta ERD is itself also very interesting, particularly since the beta ERD has previously been shown to be sensitive to response uncertainty (Tzagarakis et al., 2010). However, it is important to note the substantial differences in experimentation, and thus in interpretation, between this previous study and our current one. Perhaps most importantly, the task used by Tzagarakis et al. manipulated response certainty on a trial-by-trial basis, using a task where participants were shown pre-movement cues that provided varying numbers of potential movement options. By examining beta frequency neural activity over somato-motor regions after the onset of the cue, they found that increasing response uncertainty, as manipulated by an increase in the number of potential movement options, elicited a weaker beta ERD. This key difference in experimental design effectively focused the study on the effect of response uncertainty as it applies to the motor plan of movements that are certain to be performed. In other words, this work manipulated response certainty at the level of *response selection*. In contrast, our study manipulated the *contextual response certainty* of the motor output by altering the likelihood that a movement would be performed, while holding the movement itself constant.

Another previous study (Isabella et al., 2015) also showed modulation of motor neural oscillations, and of particular interest, of the MRGS, by contrasting these neural dynamics across cognitive “switch” and “non-switch” conditions. The current study complements these previous findings, which reported increased MRGS amplitude on trials where a second response option was required. Isabella et al. (2015) interpret these differences as an effect of motor *automaticity*, which aligns well with our findings and interpretations. Essentially, it could be hypothesized that the differences in MRGS amplitude previously reported across numerous cognitive contrasts reflects the importance of this neural response for executing less certain/automatic motor actions, relative to those that are more stereotyped and predictable. Our current work adds credence to this conceptualization, as the (un)certainty effect in this study was purely cognitive in nature (i.e., the movements being performed were identical). Interestingly, it is likely that this function of the MRGS is tightly linked to subsequent muscular function in the periphery, as gamma-band cortico-muscular coherence has been found to be selectively enhanced in anticipation of an unpredictable cue to move (Schoffelen et al., 2011).

Before closing, it is important to acknowledge the limitations of our study. First and foremost: although we found Bayesian evidence for the null hypothesis of no effect of motor certainty on the beta ERD, this should be interpreted in the context of our experimental confines. Since the unimanual trials only occurred 20% of the time, it is possible that a higher prevalence of these trials would make the motor response more uncertain, and could then potentially have a significant impact on these responses. Additionally, due to the nature of this experiment, which required the contrasting of bilateral neural responses within each participant, only responses from the primary motor cortices could be considered. The oscillatory dynamics supporting movement have repeatedly been found to extend across a distributed motor network (Heinrichs-Graham and Wilson, 2015; Wiesman et al., 2020; Wilson et al., 2014; Wilson et al., 2010), and so future studies might interrogate the effect of motor certainty on these secondary responses. This experimental design also did not allow us to effectively examine the impacts of hand dominance on these motor certainty effects, which would be an interesting avenue for future research. Finally, while a bimanual task allowed us to notably reduce the effects of inter-participant variability on our contrasts of interest, it is possible that unimanual response certainty on a bimanual task differs from unimanual response certainty on a unimanual task. While we were unable to generate any reasonable hypotheses for why this would be the case, it should still be considered in future work in this area.

Despite these caveats, the findings of this study provide new support for the MRGS playing a critical role in the active adaptation of movement execution responses to fit changing environmental contexts. This extends our current knowledge of the rhythmic neural dynamics that support adaptive mobility in humans, and might be useful in guiding targeted interventions or therapies in the future.

## Supplementary Material

1
