# Supplementary material for: Response certainty during bimanual movements reduces gamma oscillations in primary motor cortex
Source: Neuroimage. Author manuscript; Available in PMC 2021 Mar 26. (PMC7994913; doi:10.1016/j.neuroimage.2020.117448)
Supplement: 1 [file NIHMS1658697-supplement-1.pdf]

Supplementary Materials

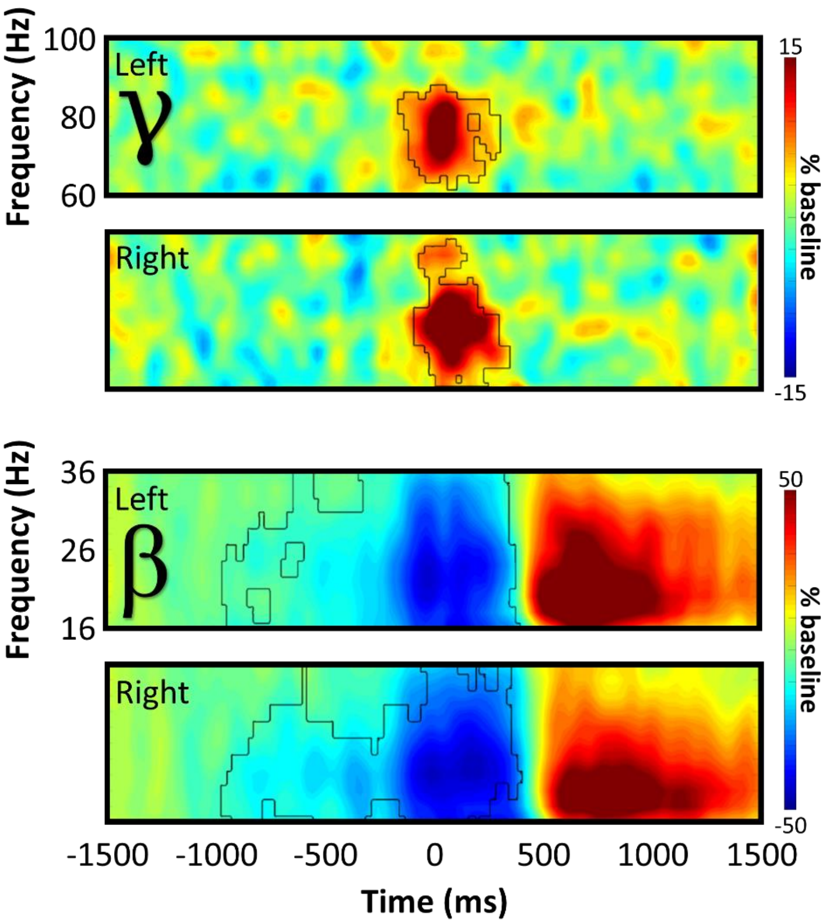

Figure S1. Time-frequency definitions of significant clusters identified in the sensor-level permutation testing.
